# Blood Stage *Plasmodium falciparum* Exhibits Biological Responses to Direct Current Electric Fields

**DOI:** 10.1371/journal.pone.0161207

**Published:** 2016-08-18

**Authors:** Lorena M. Coronado, Stephania Montealegre, Zumara Chaverra, Luis Mojica, Carlos Espinosa, Alejandro Almanza, Ricardo Correa, José A. Stoute, Rolando A. Gittens, Carmenza Spadafora

**Affiliations:** 1 Center of Cellular and Molecular Biology of Diseases (CBCMe), Instituto de Investigaciones Científicas y Servicios de Alta Tecnología (INDICASAT AIP), City of Knowledge, Panama, Republic of Panama; 2 Department of Biotechnology, Acharya Nagarjuna University, Guntur, 522 510, A.P., India; 3 School of Biotechnology, Facultad de Ciencias de la Salud “William C. Gorgas”, Universidad Latina, Panama, Republic of Panama; 4 National Center for Metrology of Panama (CENAMEP AIP), City of Knowledge, Panama, Republic of Panama; 5 Department of Medicine, Division of Infectious Diseases and Epidemiology, Pennsylvania State University College of Medicine, Hershey, Pennsylvania, United States of America; 6 Center for Biodiversity & Drug Discovery (CBDD), INDICASAT AIP, City of Knowledge, Panama, Republic of Panama; Food and Drug Administration, UNITED STATES

## Abstract

The development of resistance to insecticides by the vector of malaria and the increasingly faster appearance of resistance to antimalarial drugs by the parasite can dangerously hamper efforts to control and eradicate the disease. Alternative ways to treat this disease are urgently needed. Here we evaluate the *in vitro* effect of direct current (DC) capacitive coupling electrical stimulation on the biology and viability of *Plasmodium falciparum*. We designed a system that exposes infected erythrocytes to different capacitively coupled electric fields in order to evaluate their effect on *P*. *falciparum*. The effect on growth of the parasite, replication of DNA, mitochondrial membrane potential and level of reactive oxygen species after exposure to electric fields demonstrate that the parasite is biologically able to respond to stimuli from DC electric fields involving calcium signaling pathways.

## Introduction

Half of the world population is at risk of contracting malaria, and about half a million people die every year from this deadly disease. Of the five species responsible for the more than 214 million human infections in 2014, *Plasmodium falciparum* is startlingly credited with about 438,000 deaths [1]. Malaria has affected humans for more than two millennia and yet, despite the many advances in the understanding of the biology of the pathogen, the development of an effective vaccine has been an elusive goal, with lower than desired protection, or difficulties in its manufacture [2]. In addition, the daunting issue of drug resistance haunt malariologists worldwide and has forced the search for new therapeutic combinations to slow down its appearance [3].

Some forms of energy (e.g., heat, light) have been used by humans to treat diseases for centuries. Electrical technology has been available since the 19th century, but it has already been tested and used in various forms and numerous aspects of the medical field, with several review papers available in the literature [4–5]. The musculoskeletal field was one of the first fields to apply electrical stimulation for bone healing, after the discovery in the 1950s of the piezoelectric properties of bone [6–7]. Currently, several electrical stimulation devices are available in the clinic to treat bone non-unions and other complications with good success rates [8]. Other applications of electrical stimulation in medicine focus on the destructive, rather than regenerating, capabilities of this energy, such as pulsed electrical stimulation to avoid skin scar formation by causing irreversible damage to cell membranes of fibroblasts through electroporation [9] and eradication of biofilms from medical devices to reduce resistance to antibiotics and antimicrobials through the irreversible damage of the microorganism [10]. Over the last 40 to 50 years, the bioelectromagnetism field has established that different cell types, such as mamalian cells and bacteria, respond to electrical stimulation and this response is specific to the electrical parameters used: certain voltages can enhance growth dynamics, while other voltages can inhibit growth or actually cause death of the cells [11–12].

Landmark mechanistic studies by Nelson et al. identified Ca^2+^ signaling as a key pathway for the effects of electrical stimulation, and this signal transduction is particular to the specific type of physical stimulus applied [13]. Most *in vitro* studies conclude that electric fields can be used as biophysical stimulants, and that responses can be selected by manipulating parameters such as frequency, voltage, composition of the cell culture medium or nature of the field applied [14–15].

Electrical stimulation effects have been studied in multiple cell types like neurons, osteoblasts, epithelial cells, human keratinocytes, and human mesenchymal stem cells [16–18] but there is no literature on the effects of electrical stimulation in *Plasmodium* parasites. With the clear need to find new alternatives against the malaria-causing agent, our group set out to study the effects of different forms of energy on the malaria parasite to find parameters that could help kill or decrease the infecting capability of the parasite. Here we present for the first time that the intraerythrocitic form of *P*. *falciparum* is able to respond to external electric fields, with calcium ions mediating this response.

## Materials and Methods

### Materials

All reagents were purchased from Sigma-Aldrich (St. Louis, MO, USA) unless otherwise specified.

### Parasites and cultures

The malaria parasite strain HB3 of *P*. *falciparum* was cultivated following the method described by Haynes *et al*. [19] with some modifications. In brief, we used O+ erythrocytes in a complete medium that consisted of RPMI 1640 supplemented with 25 mM HEPES, 0.2% sodium bicarbonate, and 10% serum. Blood was obtained from blood banks, which are obtained as part of the voluntary donor act under Panamanian standard of care and scheduled for discard because of comorbid conditions of the donors. Cultures were maintained at 37°C in a gas mixture of 5% CO_2_, 5% O_2_ and 90% N_2_ at a 2% hematocrit and synchronized with alanine and thermal cycling as described by Almanza et al [20]. Trophozoite or early schizont-synchronized cultures with 1% parasitemia were seeded in 96-well plates.

### Electrical stimulation system

**Fig 1:**
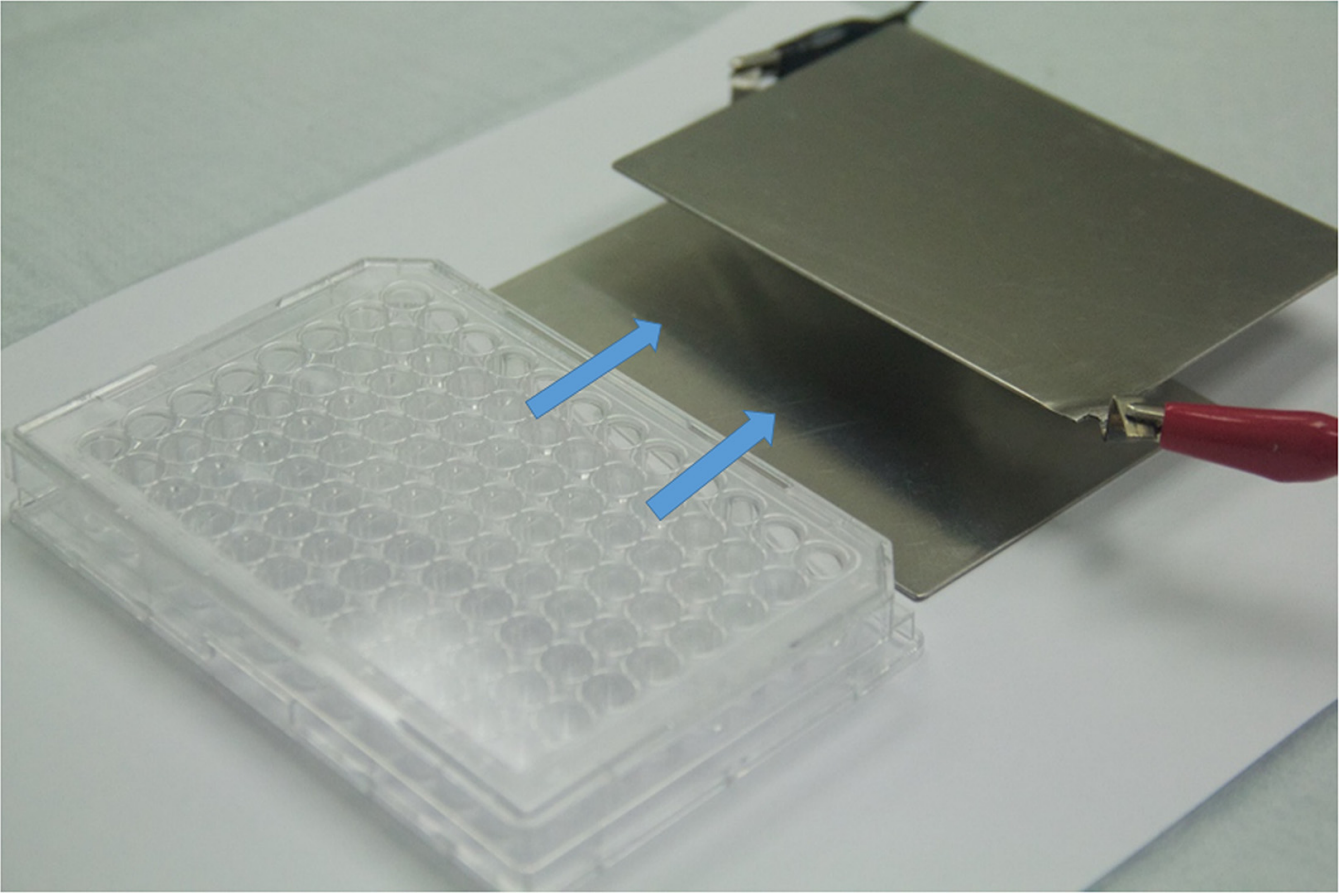
Capacitive coupling electrical stimulation system. Two stainless steel plates were designed to cover all the wells of a 96-well plate without interfering with their stacking capability. A small protruding strip on each plate was used for electrical connections.

**Fig 2:**
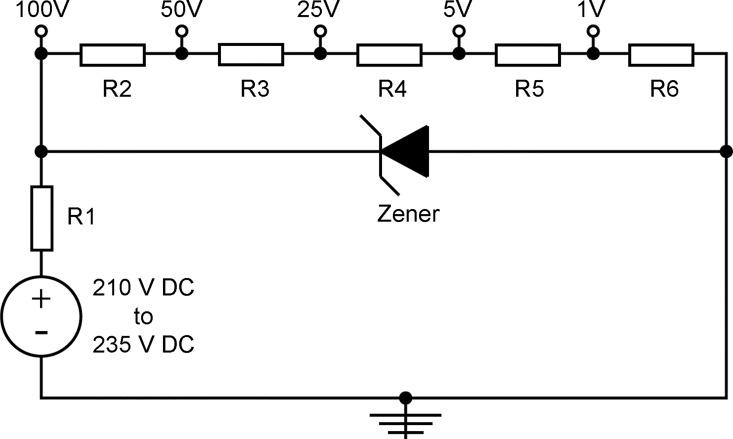
Schematic depiction of the electrical connections of the Zener-diode voltage divider. This arrangement provided the 1 V and 100 V outputs for the electrical stimulation of infected erythrocytes.

A simple *in vitro* electrical stimulation system was designed to provide direct current (DC) capacitively-coupled electric fields to *P*. *falciparum* cultures in 96-well tissue culture plates. The system consisted of two stainless steel (type 304) plates (7.8 cm x 11.3 cm x 1 mm) with a small protruding strip for electrical connections. The plates were designed to provide a capacitive electric field to the samples by sandwiching the plate and covering all wells in a standard tissue culture plate, while being used inside the incubator without interfering with the stacking of the plates (Fig 1). The voltages used in the different experiments were delivered by an EC105 DC power supply (Thermo Scientific, Waltham, MA, USA). To have better control over the voltages provided by the power supply, and to allow for different stimulation voltages at the same time, we designed a voltage divider with 5 outputs (Fig 2). The DC voltage levels provided were: 100 V, 50 V, 25 V, 5 V and 1 V. The different voltage levels were obtained from the DC power supply using a 100 V Zener diode and a set of resistors in voltage divider arrangement. A DVM 3458A high-performance digital-volt multimeter (Keysight Technologies, Santa Rosa, CA, USA) was used to calibrate and validate the outputs of the DC power supply and the voltage divider. To standardize the exposure settings for all the experiments, different times of exposure (S1 Fig) and voltages (S2 Fig) were tested in search of those with more prominent differences with respect to the controls. After these tests, 90 min of stimulation and two voltages (1 and 100 V) were chosen for all the assays.

### Parasitemia

#### Flow cytometry

After electrical stimulation, parasites were stained with 2 μg/ml Hoechst 33342 (Invitrogen, Carlsbad, CA, USA) prior to fixation with 4% paraformaldehyde, or incubated further at 37°C for 5 or 24 h before staining. Samples were stored at 4°C until analysis. A CyFlow Space cytometer (Sysmex Partec, Görlitz, Germany) was used for analysis by exciting the samples through the UV laser. The data was analyzed with the FCS Express 4 (De Novo Software, Glendale, CA, USA).

#### Microscopy

Glass slides were prepared with smears of the culture at 0, 5 and 24 hrs, after exposure, as well as with naive erythrocytes. They were fixed with methanol and stained with 20% Giemsa for 10 min. The microscopist was blinded to the treatment and at least 1000 cells were counted to determine the percent of erythrocytes infected with *P*. *falciparum* parasites.

### Characterization of the physicochemical and biological effects on controls

Naive erythrocytes and those infected with *P*. *falciparum* parasites were either not stimulated (hereinafter referred to as “Control”) or stimulated using 1 V (hereinafter referred to as “1V”) or 100 V (hereinafter referred to as “100V”) for 90 minutes, and evaluated further. Naive erythrocytes were included in our evaluation to understand if erythrocytes by themselves could respond to electrical stimulation. Control cultures were placed in the same incubator as the electrically stimulated cultures but in a separate gas-controlled chamber in order to avoid any interference from the electric fields. Control cultures were placed in the same incubator as the electrically stimulated cultures but in a separate gas-controlled chamber in order to avoid any interference from the electric fields. The pH of the cultures was measured immediately after exposure. The temperature of the culture media was also monitored to look for changes with respect to the controls. The viability of stimulated naive erythrocytes was tested 48 h later by means of assessing their subsequent capacity to sustain infection by *P*. *falciparum*. Furthermore, the hemolysis of erythrocytes after treatment was evaluated by detecting the absorbance of the media at 415 nm while using Triton X-100 as a positive control. Changes in morphology were inspected by light microscopy after Giemsa staining. Smears were taken immediately after treatment and 5 and 24 h later. As another control to test the safety of the DC electrical system under the parameters used in this project, Vero epithelial cells were exposed to 1 and 100 V for 90 min and their viability was determined 24 h later by using the MTT assay which measures cell metabolic activity.

### DNA replication

The replication of the DNA was assessed by fluorescent labeling of double stranded DNA as described previously using 50 nM chloroquine as a negative control for augmentation of DNA content [21]. Briefly, controls and electrically-stimulated groups were incubated at 37°C until the parasites reached a mature schizont stage, verified by microscopy, approximately 5 hours after stimulation. This moment roughly corresponds to 32 h after invasion, when the DNA replication rate is at its highest moment, right before merozoite egress. 50 μl of a master solution of PicoGreen mix, consisting of 1% PicoGreen^®^ (Molecular Probes, Carlsbad, CA, USA), 10 mM Tris-HCl, 1 mM EDTA, pH 7.5 (TE buffer), and 2% Triton X-100 diluted with DNAse-free water were added to the parasite cultures. The plates were incubated at 37°C for 30 min in the dark and then read in a fluorometer (FL_x_ 800; Bio-Tek Instruments, Inc., Winooski, VT, USA) using KCjunior software (Bio-Tek Instruments) to analyze their DNA replication levels. Non stimulated controls were used to normalize the measurements.

### Mitochondrial membrane potential measurement

The mitochondrial membrane potential of malaria parasites was measured using the MitoProbe™ JC-1 Assay (Life Technologies, Carlsbad, CA, USA) as per manufacturer instructions. Briefly, after electrical stimulation, each sample was centrifuged and resuspended at a cell density of 1.1x10^6^ cells/ml in 200 μl of PBS. They were incubated with JC-1 at a final concentration of 2 μM for 30 min at 37°C. Cultures were washed once with PBS and analyzed by flow cytometry. The protonophore and mitochondrial membrane disruptor carbonyl cyanide m-chlorophenyl hydrazone (CCCP, 50 μM final concentration) was used as a positive control. Cultures were processed immediately after treatment. The shift of fluorescence intensity of JC-1 from red to green is an indication of depolarization.

### Reactive Oxygen Species (ROS) measurement

Intracellular ROS was measured using CM-H2DCFDA (Molecular Probes), a non-fluorescent dye that after oxidation is transformed into fluorescent dichloroflorescein (DCF). The level of ROS present was inferred through the amount of oxidized DCF [22]. *P*. *falciparum* (2% parasitemia) cultures were incubated for 30 minutes at room temperature in the dark with PBS containing 10μM CM-H2DCFDA. Cultures were washed once with PBS, resuspended in RPMI 1640 and allowed to recover for 30 minutes in a 37°C incubator. After incubation, dye preloaded cultures were subjected to electrical stimulation treatments for 90 min, followed by a 37°C incubation for 30 min. The intracellular ROS production was measured by flow cytometry with a 488 nm argon laser as light source. All experiments were repeated at least two times, by triplicates, and the results presented are representative of these experiments.

### Transduction pathways

In order to investigate the signal pathway involved in the proliferation effect exerted on the parasites by the electrical stimuli, six different inhibitors of transduction signals of growth pathways were used. W7 (0.2 μM) (SIGMA), a calmodulin antagonist; Neomycin 5 μM (SIGMA) which blocks the inositol phosphate pathway in the cell membrane; Indomethacin (4 μg/ml) (Abcam), which inhibits prostaglandin synthesis in the cell membrane; Verapamil 1μM (Abcam), which blocks voltage gated calcium channels in the cell membrane; TMB8 10 μM (Abcam) which inhibits release of Ca^2+^ from intracellular stores; and Bromophenacyl Bromide 1 μM (Abcam), which inhibits phospholipase A in the cell membrane. Most of the inhibitors were diluted in culture media. Bromophenacyl bromide and indomethacin were diluted in DMSO to provide stock solutions which were then diluted 1:10,000 in the experimental wells, making the contribution of DMSO negligible.

The concentration of inhibitors was chosen after optimization, following the rule of using the one concentration which least affected the non-stimulated control. A non stimulated control plate, containing the inhibitors, was used as control. The compounds were added to the respective groups right before electrical stimulation and left in the wells for the duration of the treatment, and for the following 24 h period, previous to evaluation. Besides, the total parasite growth was determined both in the absence and in the presence of the specific intracellular inhibitors in the cultures stimulated by DC electric fields either at 1 V or 100 V. Each electrical stimulation run was divided into three 96 well plates (untreated, 1 V, and 100 V), where each plate contained seven (7) different treatments (without inhibitor and with each one of inhibitors W7, Neomycin, Indomethacin, Verapamil, TMB8 and Bromophenacyl bromide with at least five (5) replicas of each treatment.

### Statistical analysis

Data from the characterization of the power supply are presented as the mean ± one standard deviation (SD) of five measurements performed at different time points. Data from experiments examining parasitemia response to electrical stimulation are presented as mean ± standard error (SE) for eight individual samples per variable. Data from experiments examining DNA content and ROS measurements in response to electrical stimulation are presented as mean ± standard error (SE) of three individual samples per variable. All experiments were independently repeated at least twice, and the mean results of all experiment are presented. Data were evaluated by analysis of variance, and significant differences between groups were determined using Bonferroni’s modification of Student’s t-test. A p ≤ 0.05 was considered statistically-significant.

## Results

### Characterization of the electrical stimulation system

**Fig 3:**
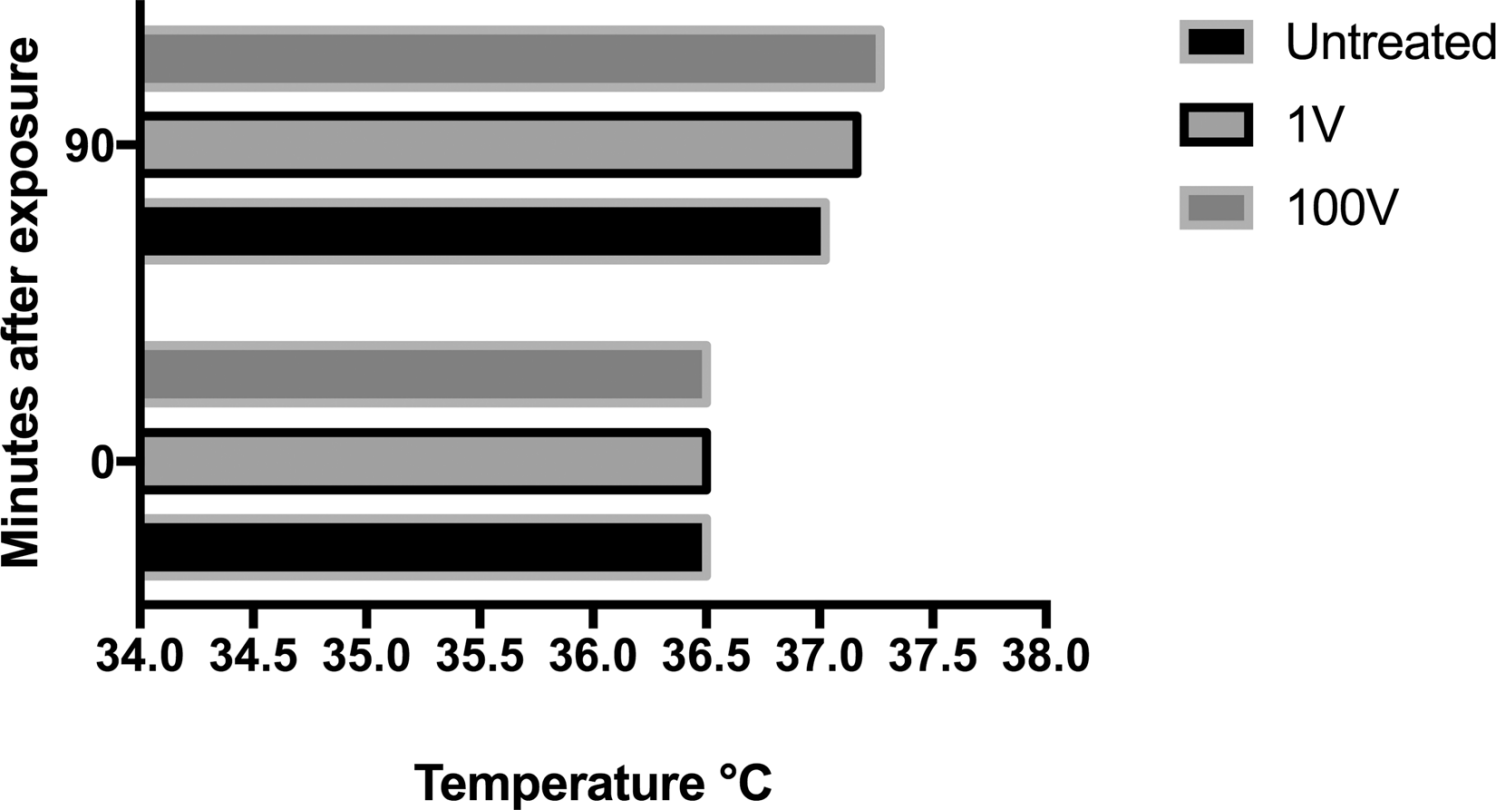
Effect of DC electrical stimulation in the temperature of the samples. The temperature of the samples was taken immediately before and after exposure. During stimulation the samples remained 90 minutes in the incubator, which was set at 37°C.

**Fig 4:**
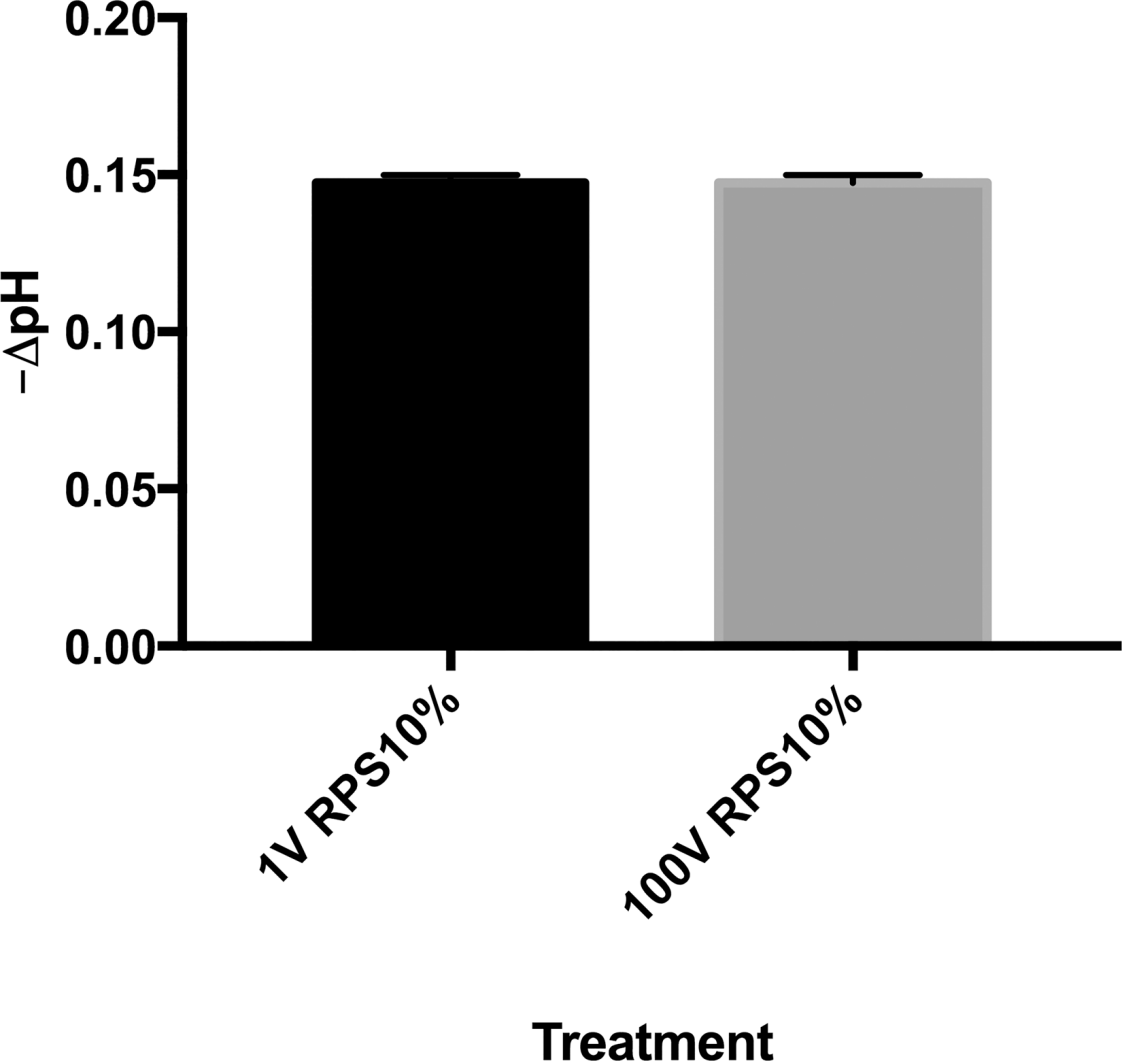
Change of pH of the culture media when exposed to DC electric fields. A mock plate with complete media (RPS) was subjected to the electrical treatment and the pH was measured before and after 90 min of exposure. The graph shows the negative change in pH produced by the exposure with respect to the initial pH.

Routine electrical characterization measurements were carried out to evaluate the performance of the power supply and the custom-designed voltage divider. In the case of the latter, the Zener diode helped ensure the output stability despite small changes in voltage that may be introduced by the power supply (S1 Table). The stability of the power supply was thus an important factor to take into account for our experiments, and an evaluation of the power source confirmed that it was within its technical specification of less than 1% of standard deviation (S2 Table).(). To rule out external effects other than the electric fields, the physicochemical parameters of the stimulation setup such as pH and temperature were assesed immediately after a simulated treatment (Fig 3). The changes in temperature between the control and treatment groups after incubation were negligible, with 37.2°C for the 1V group, 37.3°C for the 100 V group and 37.0°C for the control. The pH of the media in the treatment wells after incubation had a very modest acidification of about 0.15 units below that of the control (Fig 4).

### Biological response of naive and infected erythrocytes to electrical stimulation

**Fig 5:**
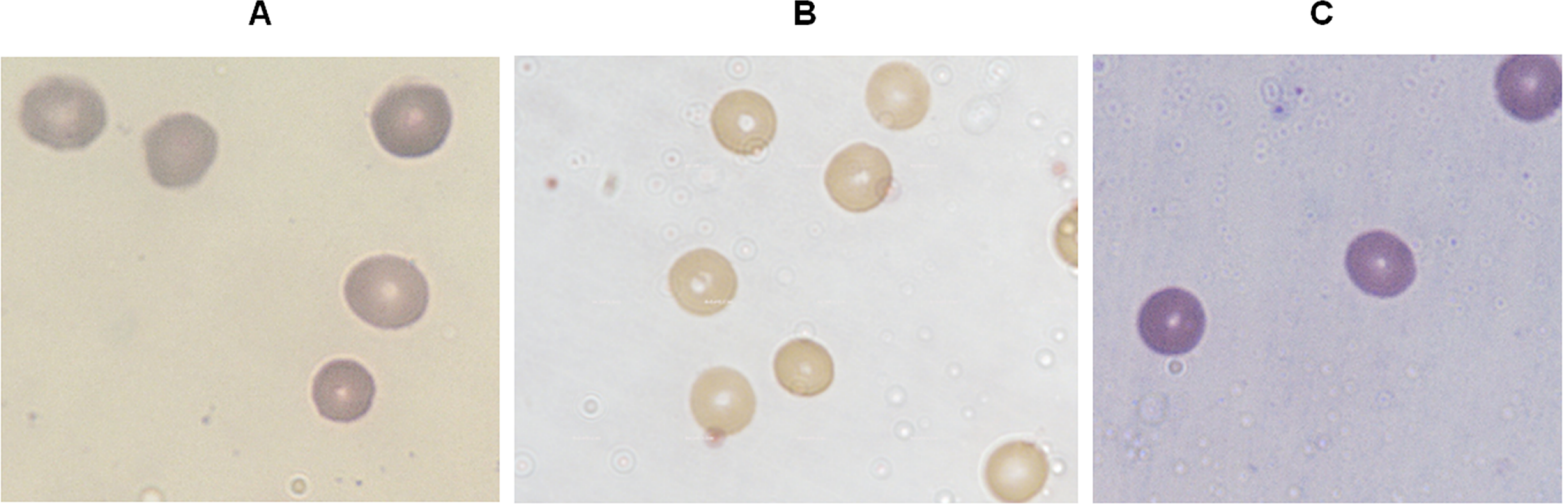
Uninfected erythrocytes exposed to DC electric fields. Naive erythrocytes (A), exposed to 1 V (B) or to 100 V (C) for 90 minutes do not show significant changes when compared to the unexposed controls. All samples were stained with Giemsa and observed under light microscopy (100 X magnification).

**Fig 6:**
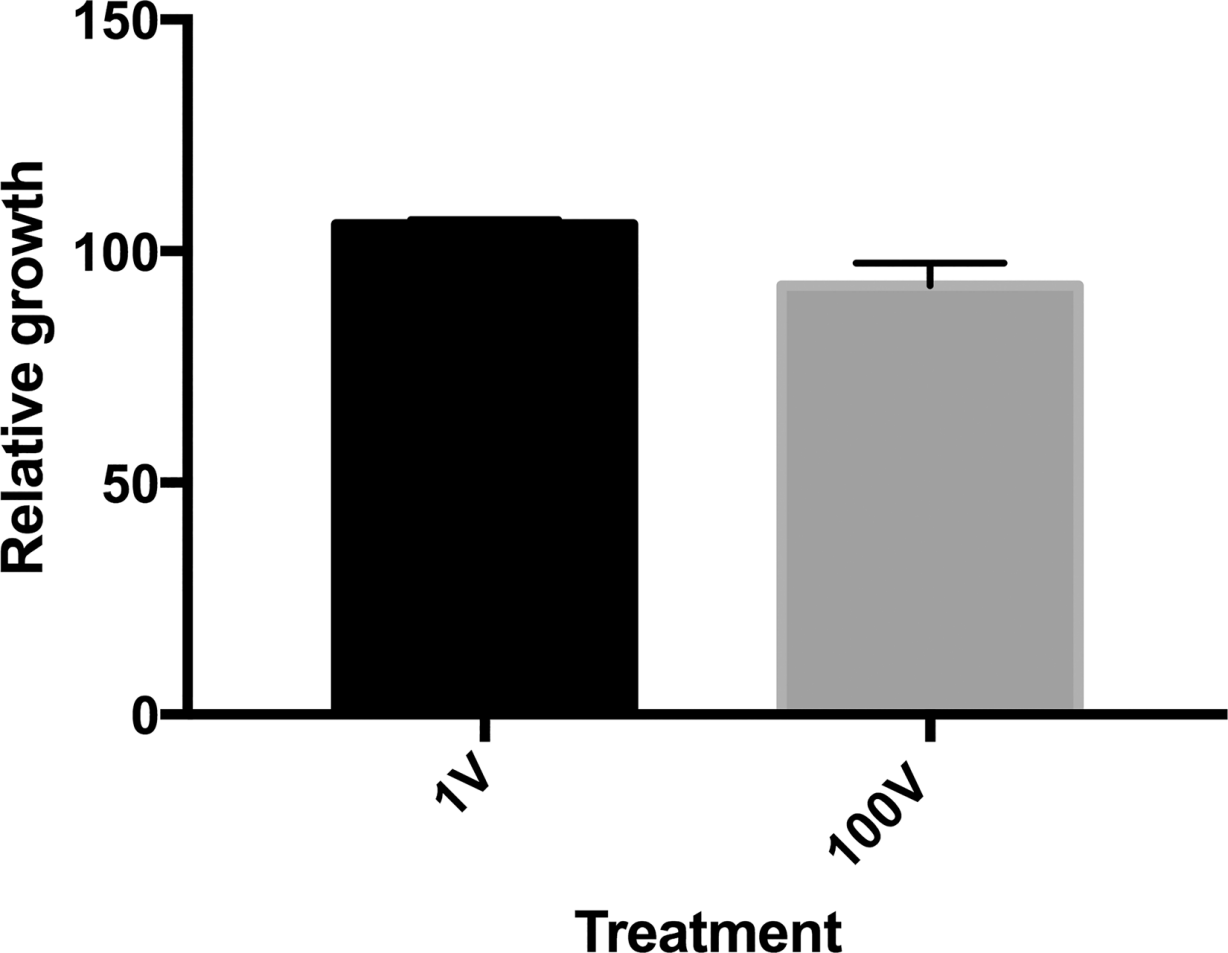
Infection by *P*. *falciparum* in erythrocytes exposed to DC electric fields. Naive erythrocytes, exposed to 100 V DC electric fields were used to culture *P*. *falciparum* parasites. The relative growth of the parasites was determined by reading the parasitemia percentage 24 hours later and comparing it to the unexposed control.

Naive erythrocytes, exposed to DC electric fields do not show evidence of responding to this treatment. The erythrocytes presented no evident changes in morphology after Giemsa staining and light microscopy observation immediatelty after exposure (Fig 5). There were no signs of significant hemolysis in the medium when subjected to light spectroscopy immediately after treatment (Table 1) nor was their viability or physiology compromised, as estimated by their capability to sustain infection by *P*. *falciparum* parasites 48 h after being exposed to these voltages (Fig 6).

**Fig 7:**
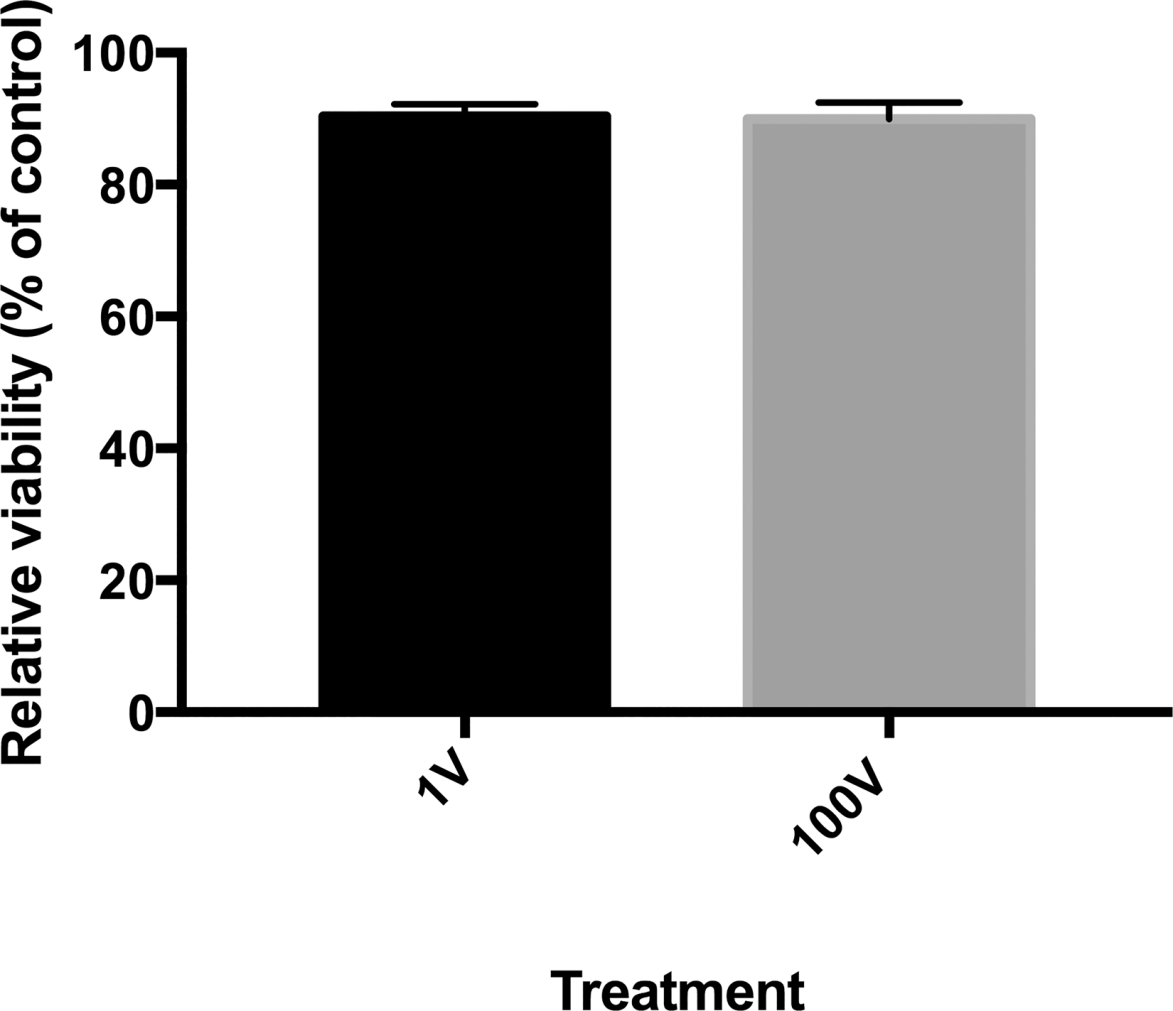
Exposure of a mammalian epithelial cell line to DC electric fields. Vero cells were exposed to capacitevely coupled DC electric fields under the same settings used to stimulate P. falciparum-infected erythrocytes. The viability of the cells was tested with the MTT assay 48 hours after treatment.

The safety of the settings to mammalian cells was tested with the Vero cell line, which also did not show any discernable loss of viability following treatment to the highest of the two exposure settings (100 V) (Fig 7)

**Table 1:** Hemolysis determination of erythrocytes exposed to DC electric fields.

Treatment	1V	100V	1% Triton X-100
**Percent lysis**	0.32	0.096	100
	0.09	0.096	100
	0.054	0.029	100

The data presented was obtained over three independent experiments. The conventional limit to determine hemolysis has been set at 1.3%

**Fig 8:**
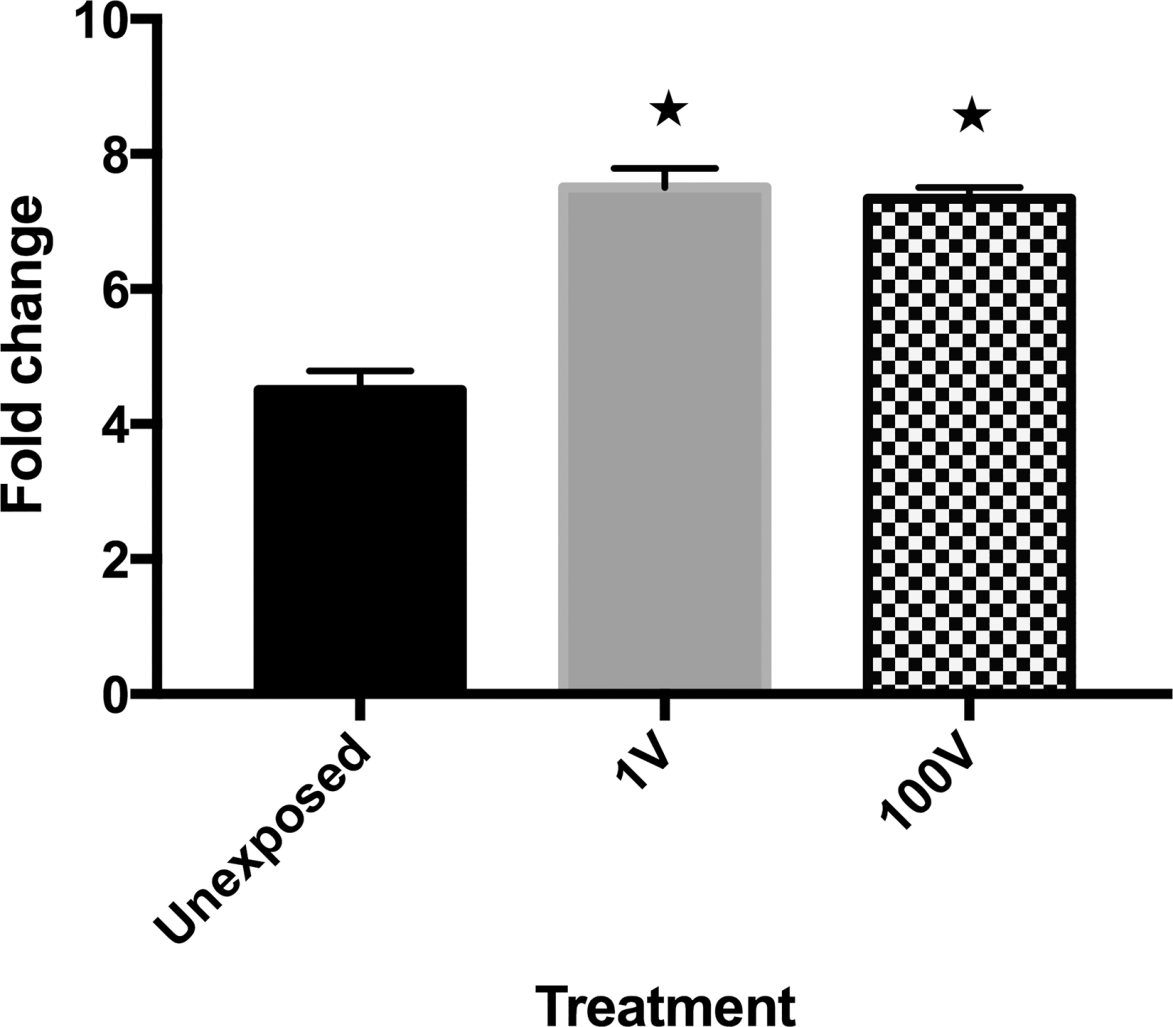
Evaluation of the fold change of growth of different experimental groups. The fold change from 0 to 24 hours was calculated as the parasitemia at 24 h divided by the parasitemia at 0 h. The data presented are the Mean ± SE of eight individual samples. One asterisk * refers to a statistically-significant difference versus control (p ≤ 0.05).

The growth of *P*. *falciparum* in infected erythrocytes *in vitro*, measured through parasitemia levels, was affected by DC electric fields. An evaluation of the parasite fold change showed that from 0 to 24 hours the 1 V and 100 V electrically-stimulated groups exhibited significantly higher growth than the controls (Fig 8).

### DNA replication

**Fig 9:**
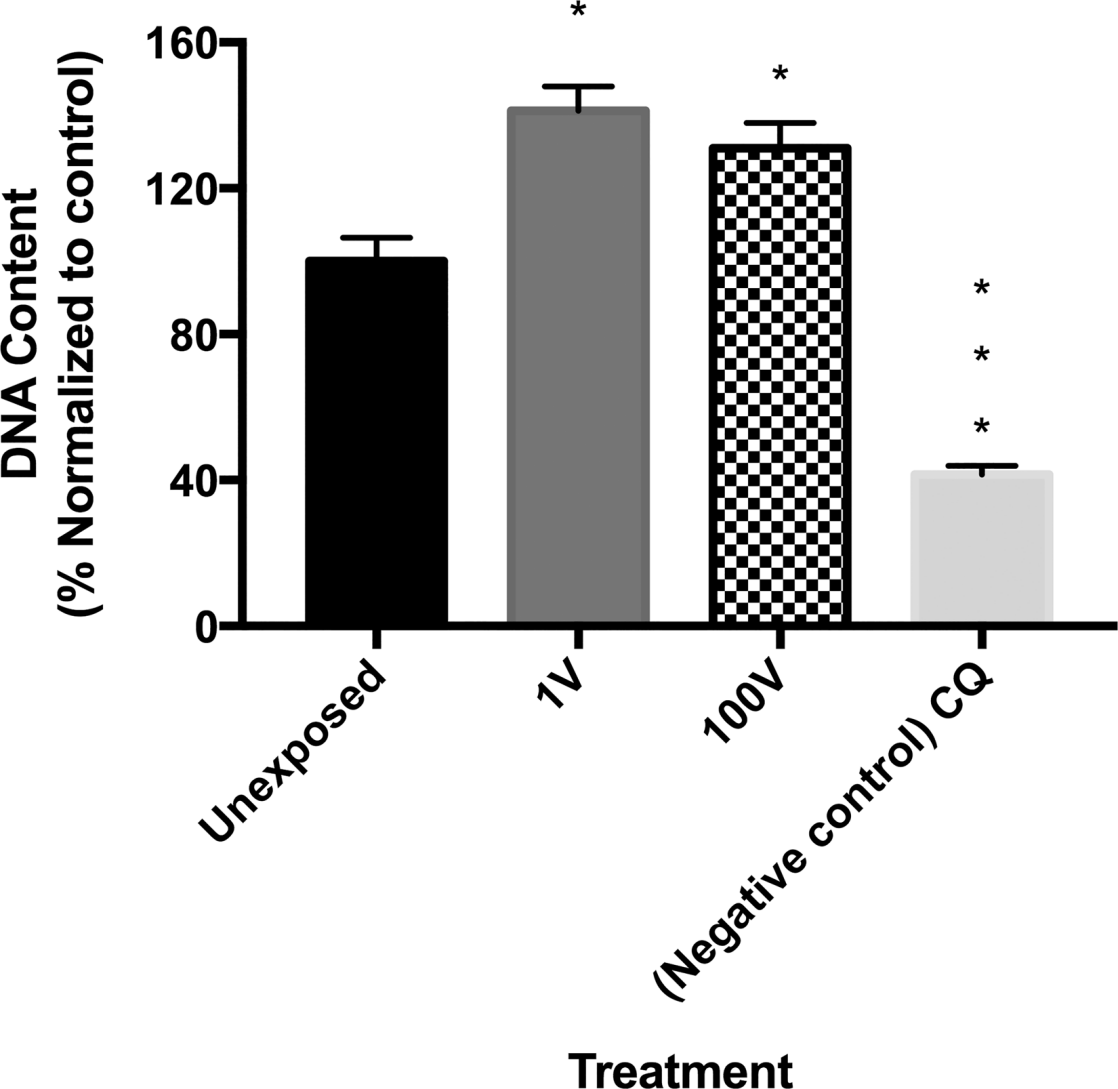
Effect of electrical stimulation on DNA replication in P. falciparum. Erythrocytes infected with P. falciparum were either not stimulated (“Unexposed”) or stimulated for 90 minutes with a capacitive voltage of 1 V (“1V”) or 100 V (“100V”) and their DNA content was measured 24 h later. Chloroquine (CQ) was used as negative control. Data presented are the mean ± SE of three individual samples per group. * Asterisks refer to a statistically-significant p ≤ 0.05 versus their respectively control exposed to 1 V and 100 V, as follows: One asterisk * p ≤ 0.01, three asterisks *** p ≤ 0.001.

The increase in parasitemia matched a significant increase in DNA content detected in infected erythrocytes exposed to the capacitive electric fields (Fig 9). The groups stimulated with 1 V and 100 V had a 1.4- and 1.3-fold increase in DNA replication levels, respectively, compared to the non-stimulated control. This highlights the positive effects of the capacitive voltage on the DNA replication capabilities of the parasite. A negative control of infected erythrocytes treated with chloroquine (CQ) exhibited an approximately 0.4-fold change in DNA replication, compared to the control, confirming the negative effect the drug exerts on the parasite.

**Fig 10:**
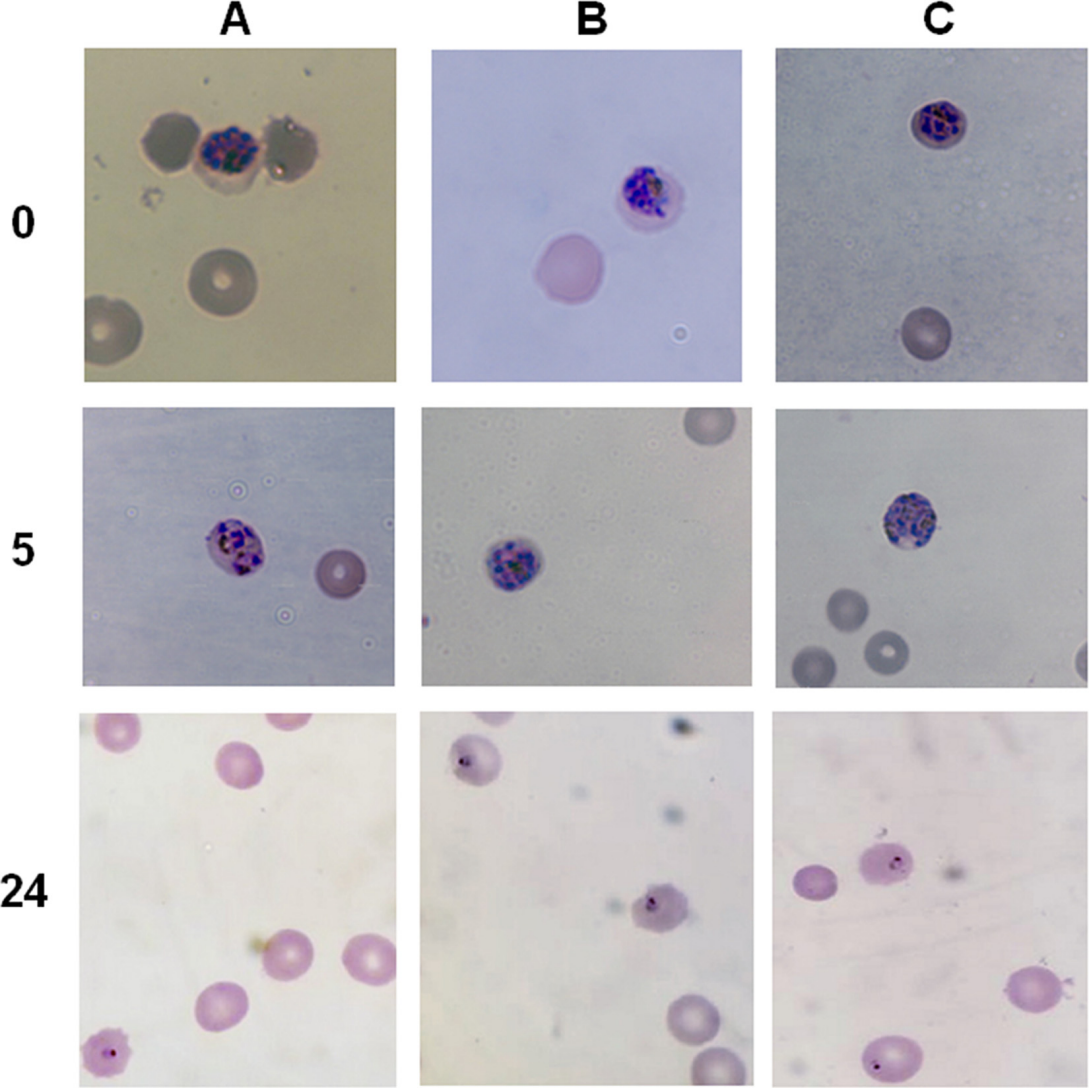
Time-series of the morphology of the intraerythrocytic parasite after DC electrical stimulation. Giemsa smears show the development of the parasites inside the erythrocytes. The left column shows the time (hours) after the different 90 min treatments: A) unexposed, B) 1 V and C) 100 V.

Optical microscopy confirms normal morphology after electrical stimulation (Fig 10). Giemsa stained smears of the parasites at 0 and 24 hours after treatment do not show any morphological disturbance when compared to controls. A smear prepared 5 hours after exposure shows that the parasites are still in a healthy schizont stage, without evidence of premature merozoite egress from the erythrocytes.

### Transduction pathways

**Fig 11:**
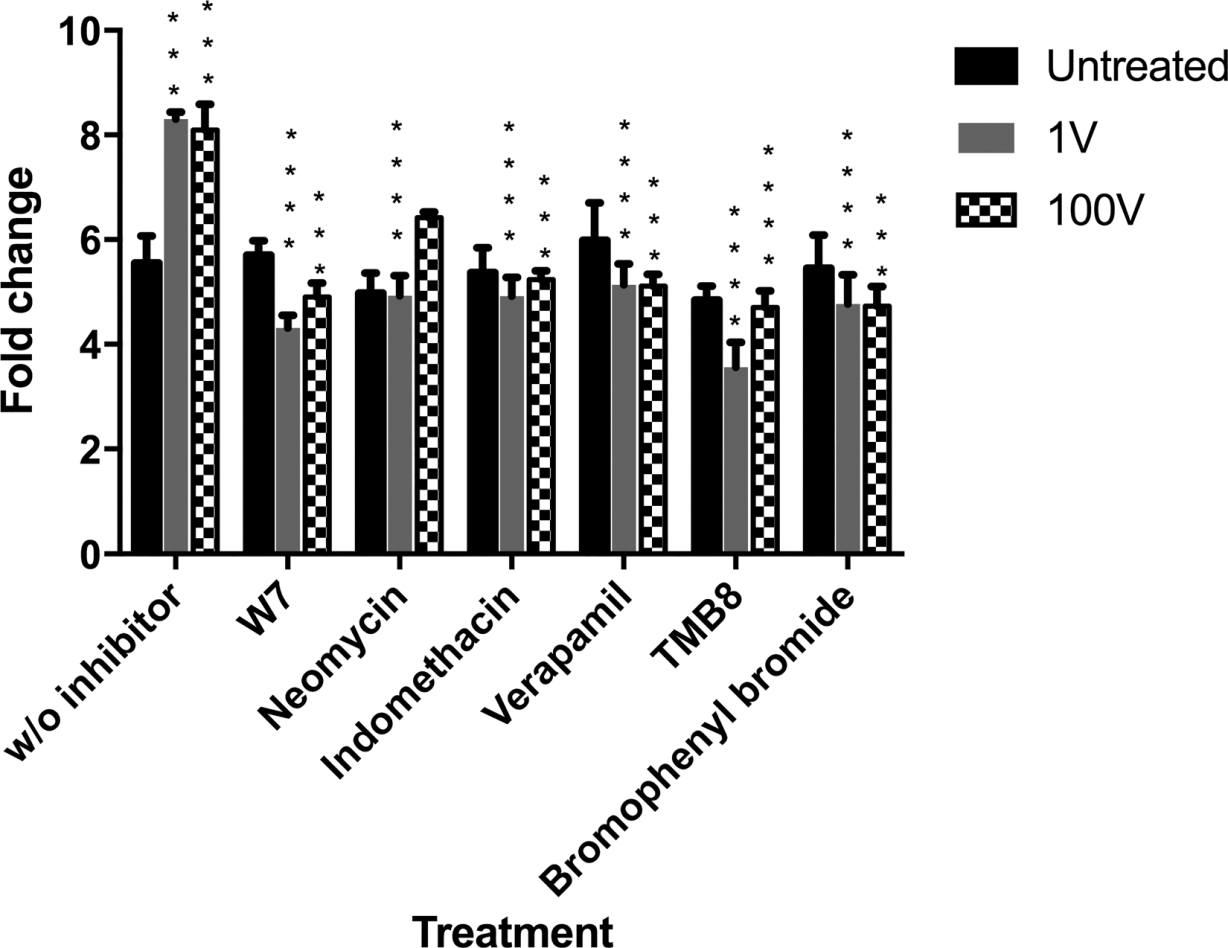
Evaluation of the fold change of growth of the different electrically stimulated groups with the use of different signal transduction inhibitors. The fold change of growth from 0 to 24 hours was calculated as the parasitemia after 24 h divided by the initial parasitemia at the time of plating. Data represented are the mean ± SE of five individual samples per group. * Asterisks refer to a statistically-significant p ≤ 0.05 versus their respectively control without inhibitor (or without treatment, for the first group of bars) exposed to 1 V and 100 V, as follows: One asterisk * p ≤ 0.01, two asterisks ** p ≤ 0.01, three asterisks *** p ≤ 0.001.

Different Ca^2+^ signaling pathways were evaluated to explore the mechanisms of action of electric fields on the malaria parasite growth (Fig 11). The increase in *P*. *falciparum* parasitemia caused by 1 and 100 V electrical stimulation, assesed 24 h later, was inhibited in a significant manner by TMB8, which blocks the release of Ca^2+^ from intracellular storages; by W7, which interferes with the activation of calmodulin; by bromophenacyl bromide and indomethacin which block the cell membrane-related pathways, either through phospholipase A or prostaglandin synthesis, and by verapamil which inhibits the uptake of calcium through L-type voltage gated channels. The only inhibitor that presented a difference between the two different voltage-stimulated groups was neomycin, which blocks the inositol trisphosphate pathway and which rescued significantly only the 100 V samples. When the next cycle of the parasite was assesed 48 h later, the proliferative effect of the stimuli had almost dissappeared and parasites were growing at a rate just a little above that of the unexposed controls, with the inhibitors not making a difference in this regard (S3 Fig).

### Mitochondrial membrane potential

**Fig 12:**
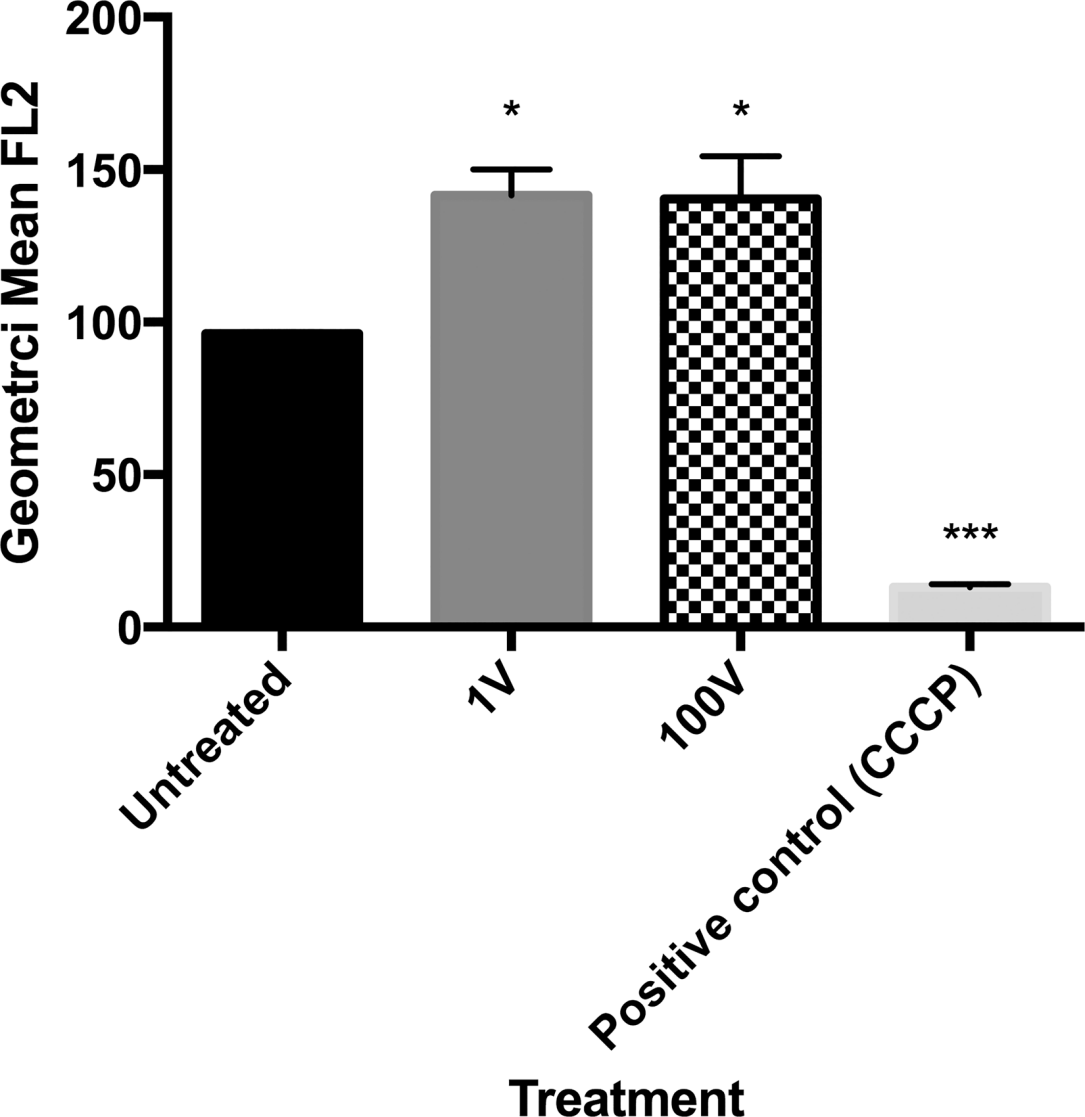
Mitochondrial membrane potential of *P*. *falciparum* after treatment with DC electric fields. Flow cytometric measurement of red JC-1 fluorescence correlates with the mitochondrial membrane potential ΔΨ_m._ Bar graphs showing the geometric mean of FL2 (red fluorescence), CCCP as positive control for mitochondrial membrane depolarization (50 μM).* Asterisks refer to a statistically-significant p ≤ 0.05 versus their respectively control exposed to 1 V and 100 V, as follows: One asterisk * p ≤ 0.01, three asterisks *** p ≤ 0.001.

To test if the proliferative response of the parasites is related to hyperpolarization of the mitochondrial membrane, as with other cell types,changes in the mitochondrial membrane potential (ΔΨ_m_) of *P*. *falciparum*, as estimated by JC-1 staining measured by flow cytometry, were assesed (Fig 12). JC-1 is a cationic dye that accumulates in the mitochondria in a potential-dependent manner. If the mitochondria were to be depolarized after exposure to the electric fields, as occurs after a detrimental challenge that commits the fate of the cells to apoptosis, a shift from green fluorescence (due to JC-1 monomers released into the cytosol) to red fluorescence (due to JC-1 aggregates inside a healthy mitochondria) should be evident in flow cytometry analyses. Infected erythrocytes were treated with a mitochondrial membrane disrupter (CCCP) as a positive control for mitochondrial depolarization. The level of red fluorescence in the unstimulated control group was used as the baseline. In the electrically-stimulated groups at 24 h, both 1 V and 100 V treatments presented a hyperpolarization of the mitochondrial membrane, by 13.9% and 16.3%, respectively.

### Reactive Oxygen Species (ROS)

**Fig 13:**
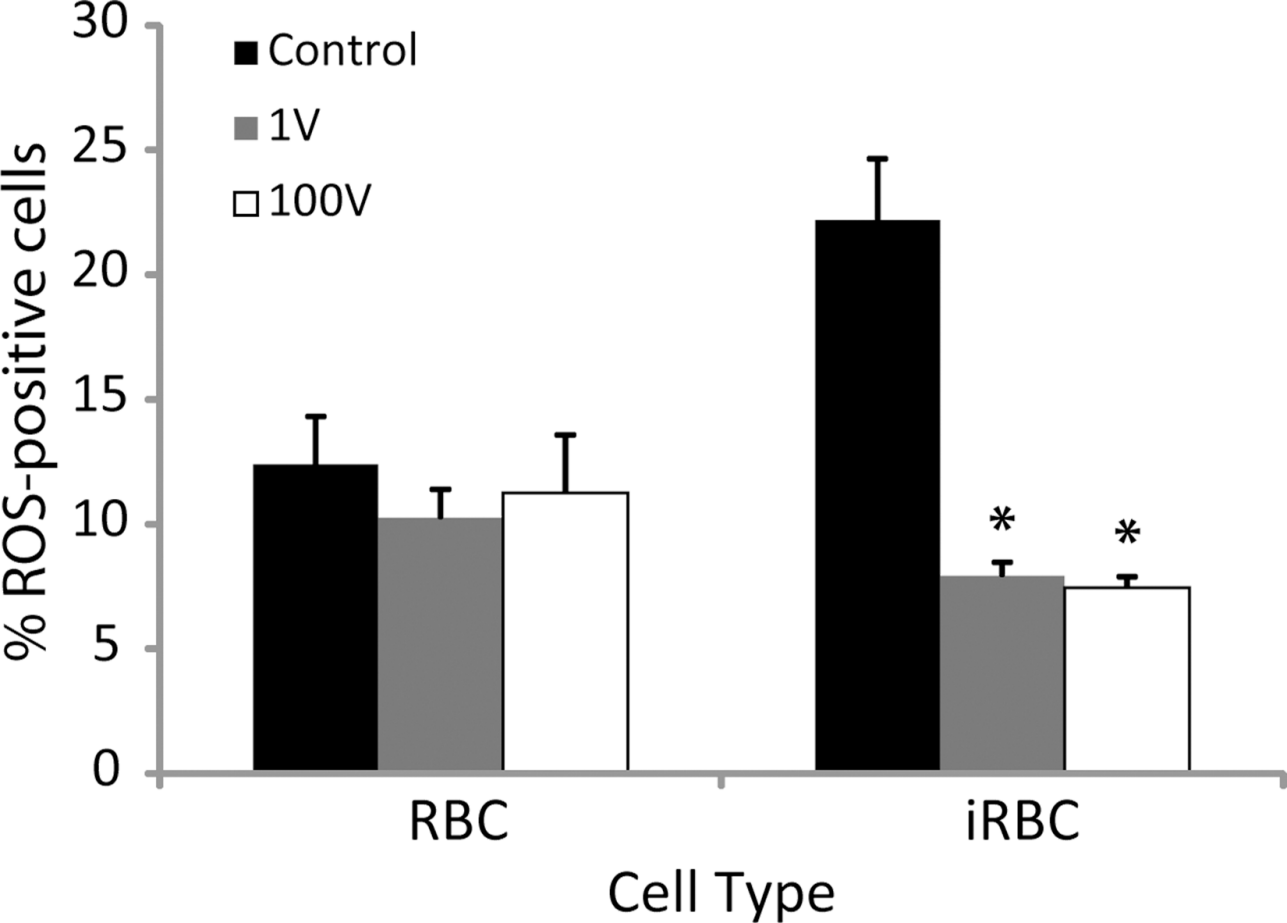
Reactive Oxygen Species produced after electrical stimulation. Healthy (RBCs) and infected (iRBC) erythrocytes were either not stimulated (“Control”) or exposed to a capacitive voltage of 1 V (“1V”) and 100 V (“100V”). The levels of ROS were measured 90 min afterwards. The graph is the mean of the results of at least two independent experiments. Data represented are the mean ± SE of 3 individual samples per group. * refers to a statistically-significant p ≤0.05 versus Control for each particular cell type.

Reactive oxygen species (ROS) are also involved in the growth of the cells, so they could be important in the proliferative response to the capacitive coupling stimulation. Compared to uninfected erythrocytes, a greater number of *P*. *falciparum* infected erythrocytes exhibited reduced levels of reactive oxygen species (ROS) after exposure to DC capacitive stimulation (Fig 13). Healthy erythrocytes were slightly affected when electrically stimulated, displaying approximately 11% increase in ROS-positive population with both voltages used.

**Fig 14:**
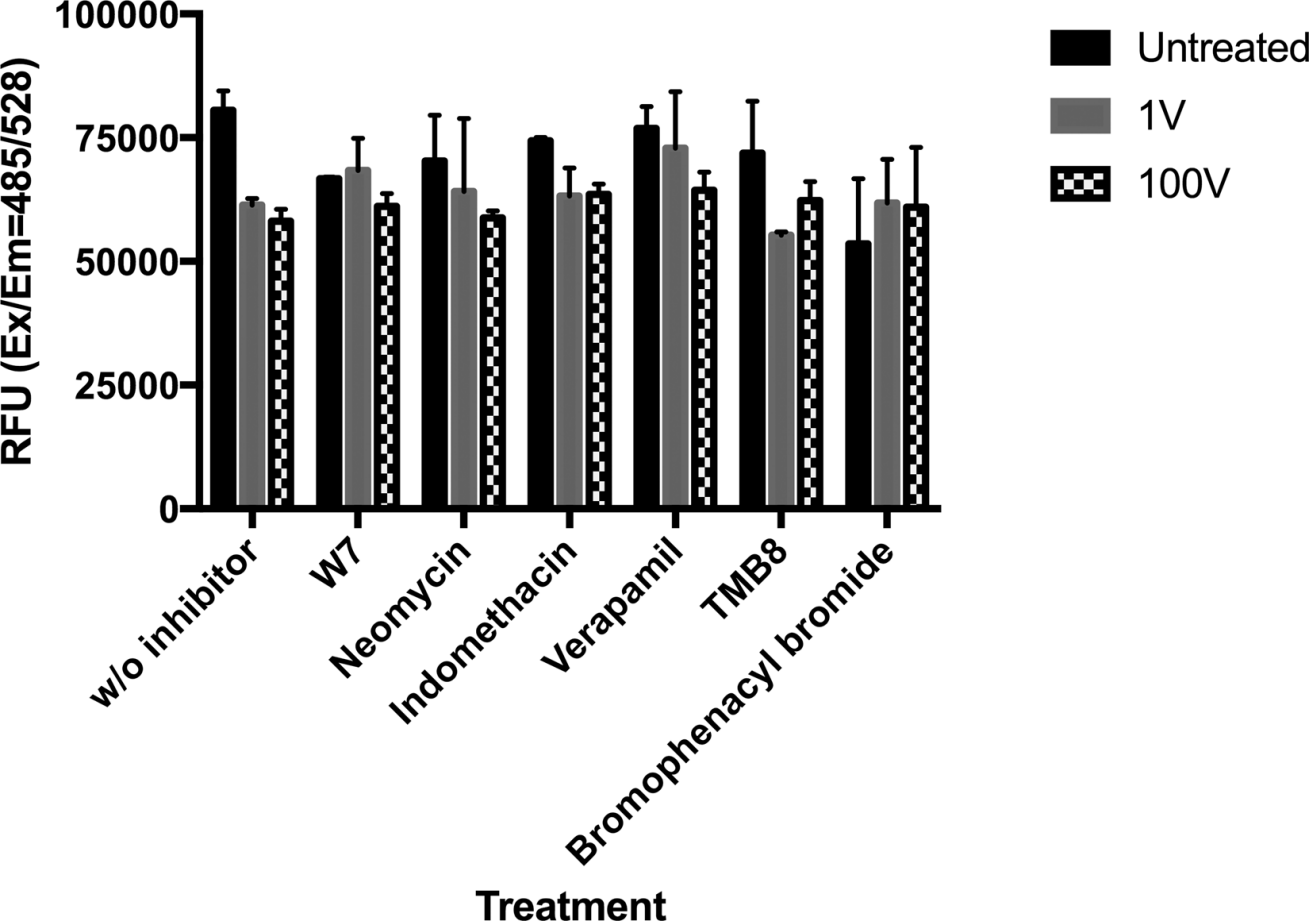
Reactive Oxygen Species. Infected (iRBC) erythrocytes were either not stimulated (“untreated”) or exposed to a capacitive voltage of 1 V (“1V”) and 100 V (“100V”), and the levels of ROS were measured immediately after the 90 min treatment. The graph is representative of the result of at least two independent experiments. Data represented are the mean ± SE of 3 individual samples per group.

In the case of infected erythrocytes, the stimulation caused almost a 3-fold decrease in the ROS-positive population compared to the non-stimulated group, with no evident difference between the two voltages applied. The use of inhibitors of different signal transduction pathways did not seem to affect this decrease in the ROS-positive population (Fig 14).

## Discussion

Every available approach, including alternative methodologies, resulting in a sustainable treatment against the malaria parasite should be pursued. We designed a system that allowed us to expose a set of samples to capacitively coupled DC electric fieds. The parameters used after standardization of the treatment do not seem to involve temperature or pH changes in the media, as they are both negligible. The exposure is not detrimental to the erthrocytes either. Assesing the viability and apparent health of the parasites, with no signs of hemolysis, asserts this. Through the experiments presented here we demonstrate that *P*. *falciparum* parasites are able to respond to external electrical stimulation.To our surprise, when late trophozoites/early schizont parasites were exposed to DC electric fields, the population of infected erythrocytes at 24 hours increased above unexposed controls. An increase in parasitemia levels in the electrically-stimulated groups could be related to higher DNA replication levels, resulting in the generation of more merozoites (invading forms) inside each schizont-stage parasite. In order to determine if a similar change in the rate of DNA replication was taking place, a nucleotide intercalator dye was added to the culture five hours after exposure to the electric fields, to coincide with the parasite’s natural highest DNA replication stage. We then measured the double-stranded DNA content of the cultures five hours after exposure and found a significant increment with respect to the control. Microscopic examination of the parasites at this moment show intact schizont parasites with no premature egress of merozoites into the media.These results coupled to the parasitemia count through Giemsa staining or by flow cytometry suggest that the elevated amount of DNA was effectively committed to complete cell cycles that resulted in an increased parasite population. In fact, healthy rings are observed by microscopy 24 h after the electric stimuli in both 1 and 100 V treated groups.

The pathways targeted by the electrical stimulation system seem to be related to the Ca^2+^ signaling cascade. Signal transduction in both the 1 V and 100 V stimuli points to activation of intracellular Ca^2+^ release channels that flood the cytosol with the ion and promote the activation of calmodulin. Stimulating with 1 or 100 V also had direct interaction with the plasma membrane, since inhibitors of voltage-gated Ca^2+^ channels, phospholipase A, and prostaglandin synthesis were able to revert the proliferative effect of the electrical stimulation. An important peculiarity we found was that neomycin, which blocks the inositol phosphate (IP3) pathway, did not cause a significant reduction of the proliferative effect in the 100 V treated group, marking a difference with the lower intensity stimulated parasites. These results are in agreement with Brighton et al. (2001) [23], who very elegantly showed for the first time that different electrical stimulation systems (i.e., capacitive-coupling, inductive-coupling and combined fields) used different Ca^2+^ signaling pathways. They found that capacitive-coupling stimulation depended mainly on voltage-gated channels to supply Ca^2+^ into the cytosol, similar to what we found with our stimulations. Interestingly, inductive-coupling and combined fields stimulation depended strictly on intracellular Ca^2+^ storage for calmodulin activation. Examining the pattern of rescue of the effect caused by the stimulus with each added inhibitor, our results point to the involvement of both extracellular as well as intracellular calcium entry into the cytosol.

The application of electric fields has previously been shown to affect the polarization of the mitochondrial membrane [24]. Thus, we analyzed the response of the parasite mitochondrial membrane potential, ΔΨ_m_, to DC electric fields. Accordingly with what had been reported, we did not see a depolarization, usually related to apoptosis processes, but a hyperpolarization of the mitochondria. This finding is in agreement with reports where proliferation events, beyond normal patterns, exhibit this type of change in the membrane of the mitochondria [25]. In fact, cancer cells show a similar hyperpolarization [26].

At present, not enough information is available to dilucidate clearly how *P*. *falciparum* responds to electrical stimulation. Ca^2+^ channels have been reported in genomic analyses of some parasites [27–28], but no canonical voltage-gated Ca^2+^ channels seem to be present in *P*. *falciparum* following this type of examination. This is at odds, however, with studies where verapamil, a voltage-gated L-type Ca^2+^ channel blocker, has seemingly affected the intake/uptake of Ca^2+^, pointing to the presence of some sort of homologue protein, maybe of a different nature, activated by electric currents in *P*. *falciparum*, which would not be surprising, given the wide genetic differences between *Plasmodium* and higher eukaryotes [29–31]. Our results coupled to our evaluations with Ca^2+^ signaling inhibitors point to a possible scenario where plasma membrane voltage-gated Ca^2+^ (or equivalent) channels are activated after capacitive stimulation, allowing for the influx of Ca^2+^ from the erythrocyte cytosol. It has been reported that *Plasmodium*-infected erythrocytes have an influx of extracellular Ca^2+^ which is 10 to 20 times higher than that of uninfected cells, especially as the parasite grows inside the blood cell [32]. Recent findings describe a Ca^2+^ uniporter and at least one mitochondrial Ca^2+^ efflux pump, PfCHA, which locates in this organelle and transports Ca^2+^ and Mn^2+^ out of the mitochondria to keep the membrane potential [33]. However, a recent report found that, under physiological conditions, Ca^2+^ influx into the mitochondria via the mitochondrial Ca^2+^ uniporter is small relative to other cytosolic Ca^2+^ extrusion pathways [34]. An excess of positive ions in the cytosol of the parasite could explain the hyperpolarization of the mitochondria. This hypothesis is supported by our finding that both electrical stimuli were able to facilitate intracellular and extracellular calcium influx into the cytosol, specially in the case of the 100 V group, with an extra contribution from the activation of IP3 signalling.

A common result of external injury to the cells is the increase in the level of reactive oxygen species (ROS), thus a decrease in ROS levels was not surprising after observing the proliferation caused by electric stimulation. It has been well established that lowering the levels of damaging oxidative species confers protection to the cells, and a number of antioxidants are used to achieve this goal such as Vitamin C, polyphenols or omega-3 fatty acids, to name just a few [35–37]. *P*. *falciparum* normally exhibits a high basal level of ROS [38]. This is due to the fact that, while in the erythrocyte, the parasite digests haemoglobin converting it into haem and globin in the food vacuole. While the parasite biocrystallizes this toxic heme into hemozoin, some free heme leaks into the cytoplasm, initiating a series of oxidation reactions that produce superoxides and hydrogen peroxide, in turn causing lipid peroxidation and DNA damage. We used the CM-H2DCFDA dye to read the level of ROS produced after DC electric field stimulation. As expected, ROS baseline levels in the non-stimulated infected erythrocytes were relatively high, but, in accordance with our previous results, we found a significant decrease in reactive oxygen species levels in the DC electrically stimulated groups as compared to the unexposed controls. The addition of inhibitors did not reverse the lowering ROS levels caused by the treatment, which probably indicates that our observed reduction of ROS is unrelated to the Ca^2+^/Calmodulin signal transduction pathway. Arguably, there is a more pronounced tendency towards a reversal with the use of verapamil, suggesting that voltage dependant calcium channels might have some more relevant contribution to the decreased amount of ROS after stimulation than any of the other receptors or second messengers studied here. Uninfected RBCs remained relatively unaltered, with relatively low ROS levels, indicating that the measured changes in ROS come from the parasite.

We aimed to study the action of DC capacitively coupled electrical stimulation by designing a setup that would allow the treatment of samples in a 96-well plate and standardized the parameters to be used with *P*. *falciparum* parasites. We found that treatment with 1 and 100 V causes an increase in the proliferation of this pathogen. Employing several signal transduction inhibitors we determined that calcium signaling appears to have a major role in the observed effects. The amount of energy delivered to the sample with the use of low and high voltage (1 V and 100 V, respectively) appears to not have important implications in the mode of action between the two treatments, except for the IP3 membrane pathway (blocked by neomicin) which seems to be used not so pronouncedly by the samples stimulated with 100 V. It is evident that stimulation with DC capacitively coupled electric fields seems to trigger a signaling pathway that involves the release of calcium from intracellular stores leading to the activation of calmodulin signaling. The increase in activation of calmodulin seems to also involve intracellular Ca^2+^ storages, as well as Ca^2+^ translocation through cell membrane voltage-dependent Ca^2+^ channels.

The mechanism of action of some antimalarials involves reactions that induce an increment in ROS production (Fe^3+^-in the case of artemisinin), depolarization of the plasma and/or mitochondrial membrane, DNA fragmentation and the final death of the parasite [39]. The mode of action of the DC electric fields applied in this study seem to be affecting this pathway in the reverse way: reducing the production of ROS, hyperpolarizing the mitochondrial membrane, and increasing the synthesis of DNA, all of which translates into higher proliferation rates and an increase in the number of parasites.

## Conclusions

The most important finding of this study is that *P*. *falciparum* is able to respond to direct current electric fields. In this work we have shown that there are biological effects associated with an exposure to this type of energetic stimulation. Our results seem to suggest that the mechanism of action of these effects involves specific signaling pathways related to calcium directly involved in the increase of mitochondrial membrane potential polarization.

We have found that specific DC electric fields help the malaria parasite to proliferate, which was not our initial expected outcome. Nonetheless, showing that the parasite is capable of reacting when confronted with electrical stimulation opens up the possibility of finding appropriate energy parameters with which to manipulate their response.

## Supporting Information

S1 Fig Standardization of time of exposure. Infected erythrocytes were exposed to 25 V DC electric fields for different times and their growth assesed 24 hours later.
(TIFF)

S2 Fig Standardization of the voltage to be applied. Infected erythrocytes were exposed to different voltages of DC electric fields and their growth assesed 24 hours later.
(TIFF)

S3 Fig Fold change of growth in the second cell cycle of the different electrically stimulated groups of parasites with the use of different signal transduction inhibitors. The fold change in growth from 24 to 48 hours was calculated as the parasitemia after 24 h divided by the initial parasitemia at the time of plating. Data represented are the mean ± SE of five individual samples per group. * Asterisks refer to a statistically-significant p ≤ 0.05 versus their respectively control without inhibitor (or without treatment, for the first group of bars) exposed to 1 V and 100 V, as follows: One asterisk * p ≤ 0.01, two asterisks ** p ≤ 0.01, three asterisks *** p ≤ 0.001
(TIFF)

S1 Table Stability of the Power Source EC105. (TIF)

S2 Table Characterization of the five output voltage levels of the resistive voltage divider at different input voltages. (TIF)
