# Supplementary figures and images for: Blood Stage Plasmodium falciparum Exhibits Biological Responses to Direct Current Electric Fields
Source: PLoS One. 2016 Aug 18;11(8):e0161207. doi: 10.1371/journal.pone.0161207 (PMC4990222; doi:10.1371/journal.pone.0161207)

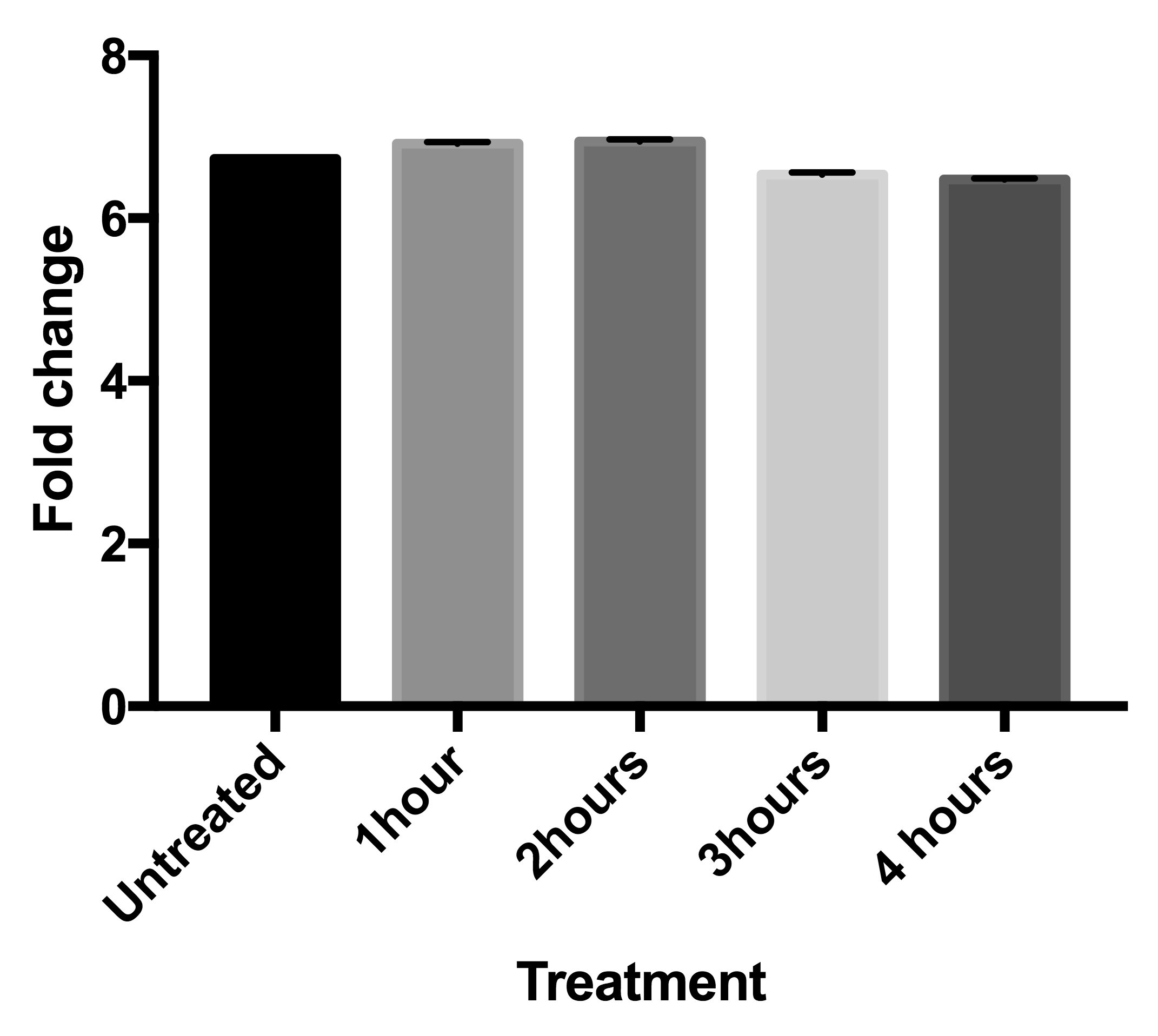

Supplement: S1 Fig — Infected erythrocytes were exposed to 25 V DC electric fields for different times and their growth assesed 24 hours later. (TIFF) [file pone.0161207.s001.tiff]

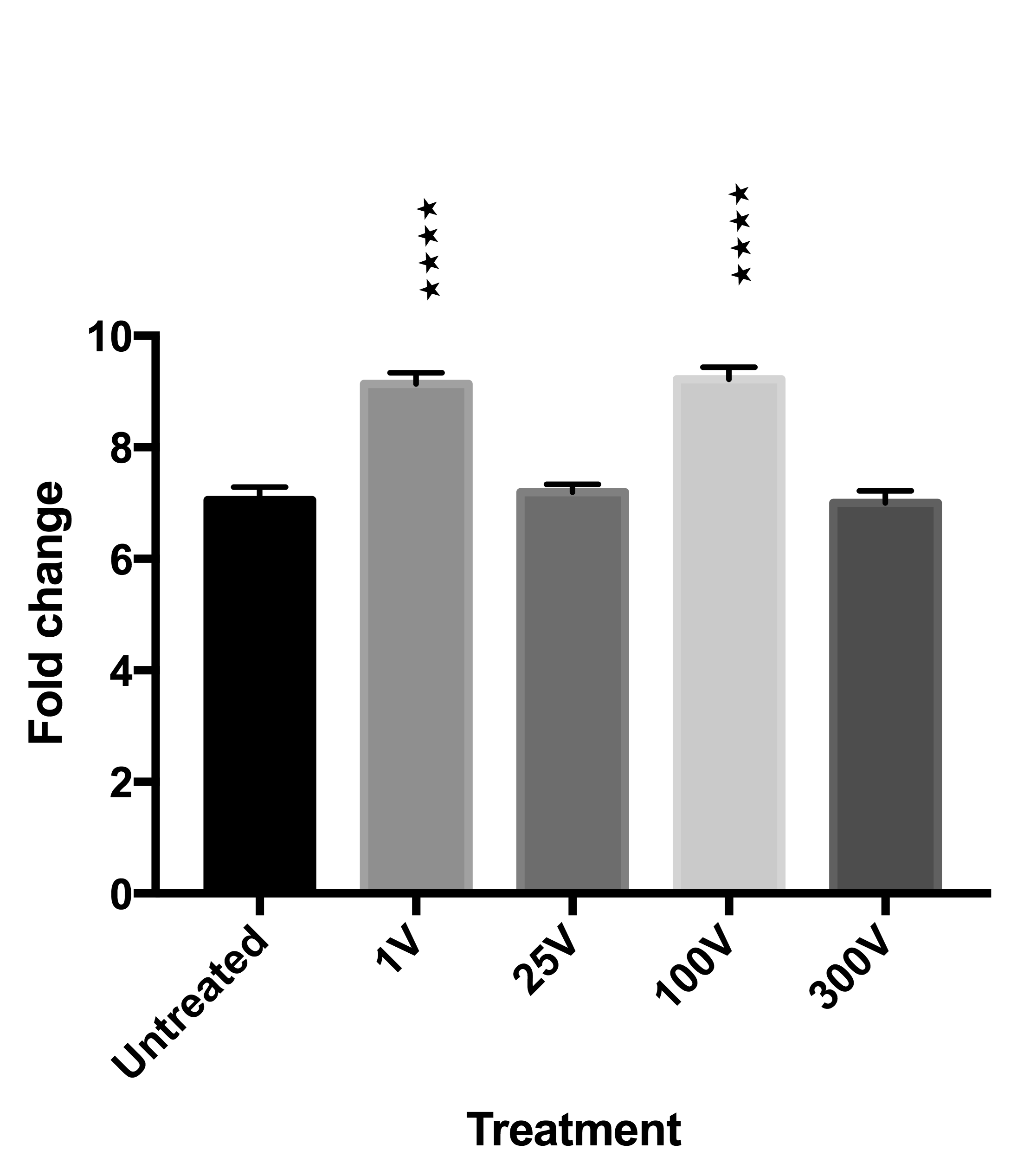

Supplement: S2 Fig — Infected erythrocytes were exposed to different voltages of DC electric fields and their growth assesed 24 hours later. (TIFF) [file pone.0161207.s002.tiff]

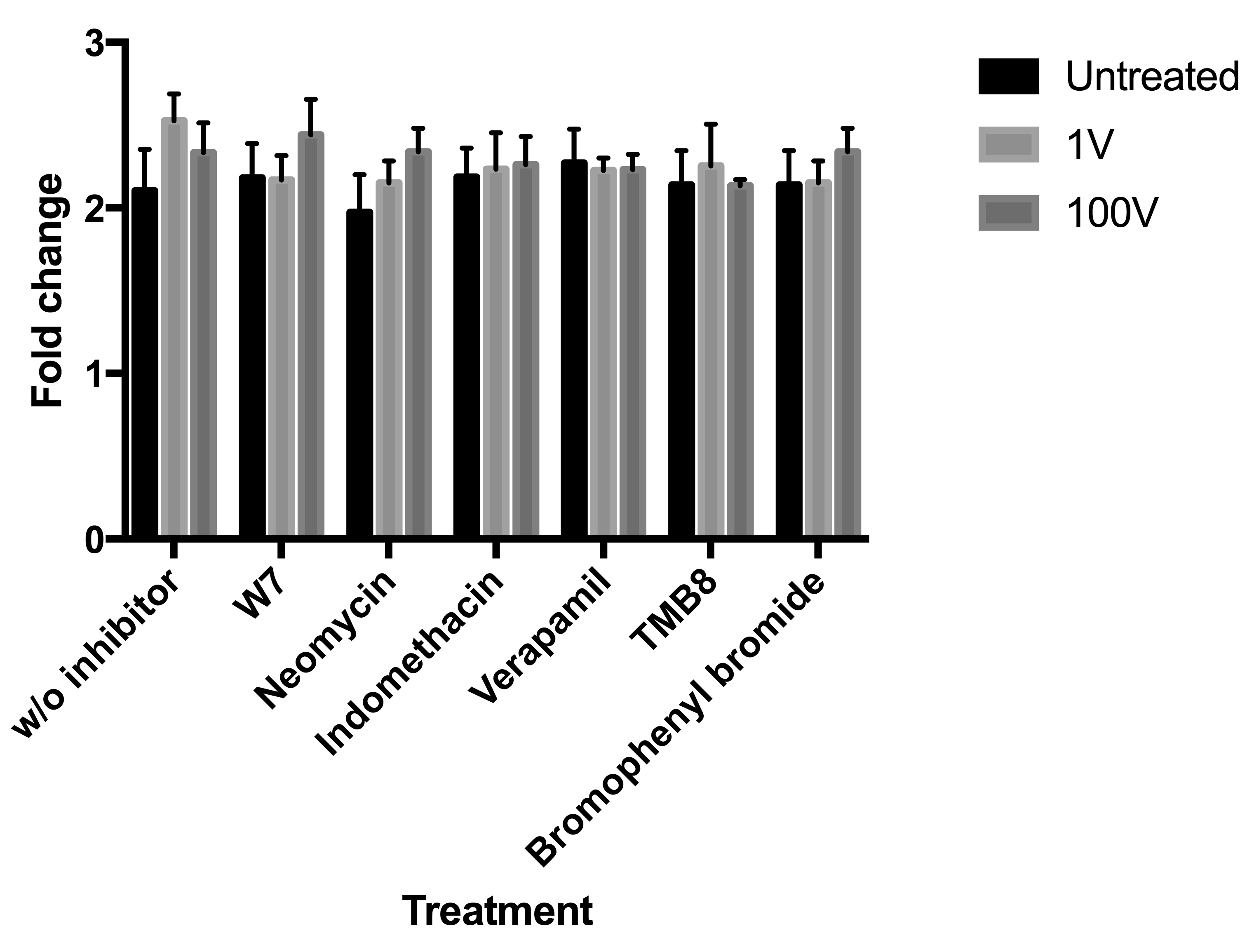

Supplement: S3 Fig — The fold change in growth from 24 to 48 hours was calculated as the parasitemia after 24 h divided by the initial parasitemia at the time of plating. Data represented are the mean ± SE of five individual samples per group. * Asterisks refer to a statistically-significant p ≤ 0.05 versus their respectively control without inhibitor (or without treatment, for the first group of bars) exposed to 1 V and 100 V, as follows: One asterisk * p ≤ 0.01, two asterisks ** p ≤ 0.01, three asterisks *** p ≤ 0.001 (TIFF) [file pone.0161207.s003.tiff]

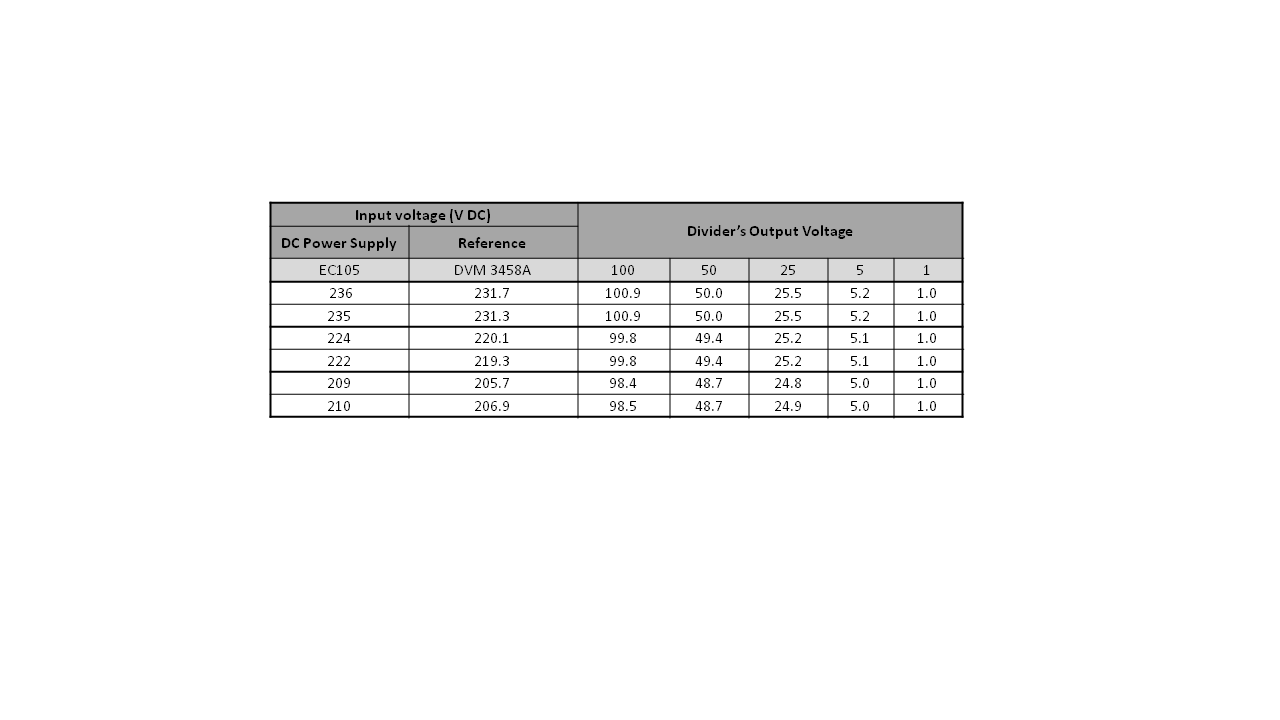

Supplement: S1 Table — (TIF) [file pone.0161207.s004.tif]

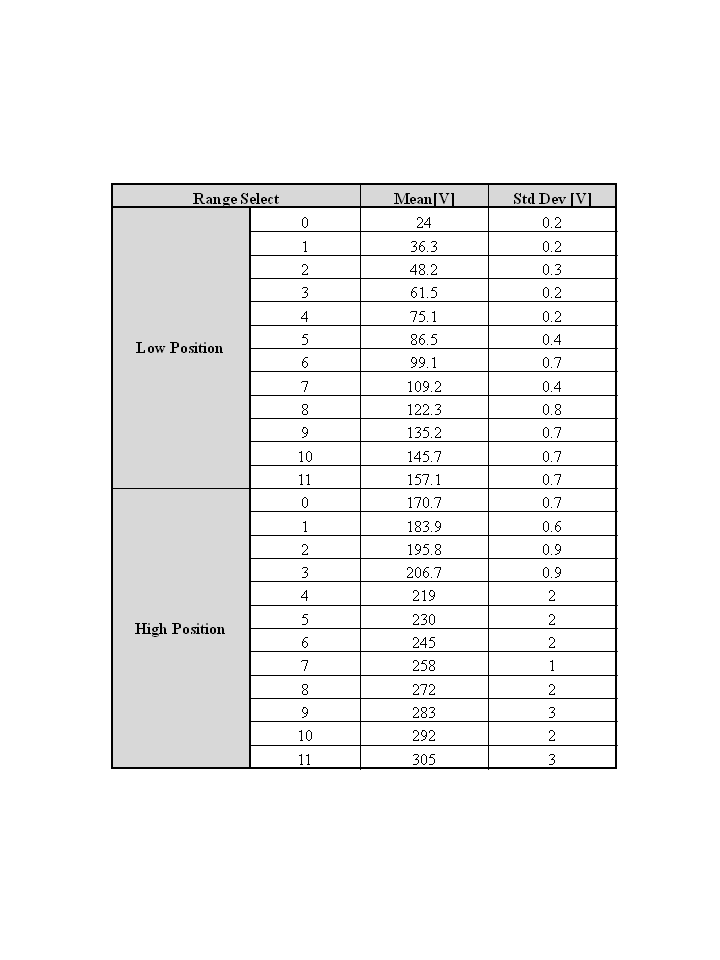

Supplement: S2 Table — (TIF) [file pone.0161207.s005.tif]
